# Serum MMP-8 and TIMP-1 predict prognosis in colorectal cancer

**DOI:** 10.1186/s12885-018-4589-x

**Published:** 2018-06-22

**Authors:** Camilla Böckelman, Ines Beilmann-Lehtonen, Tuomas Kaprio, Selja Koskensalo, Taina Tervahartiala, Harri Mustonen, Ulf-Håkan Stenman, Timo Sorsa, Caj Haglund

**Affiliations:** 1 Department of Surgery, University of Helsinki and Helsinki University Hospital, P.O. Box 105, Haartmaninkatu 4, Terkon tutkijatilat, 3. krs, FIN-00029 HUS Helsinki, Finland; 2 Research Programs Unit, Translational Cancer Biology, University of Helsinki, P.O. Box 105, Haartmaninkatu 4, Terkon tutkijatilat, 3. krs, FIN-00029 HUS Helsinki, Finland; 3 Department of Oral and Maxillofacial Diseases, Helsinki University Hospital and Biomedicum Helsinki, P.O. Box 63, Haartmaninkatu 8, 2nd floor, FIN-00014 Helsinki, Finland; 4 Department of Clinical Chemistry, University of Helsinki and Helsinki University Hospital, P.O. Box 700, FIN-00029 HUS Helsinki, Finland; 5 Department of Dental Medicine, Karolinska Institutet, Huddinge, Sweden

**Keywords:** Colorectal cancer, Colon cancer, MMP-8, MMP-9, TIMP-1, Prognosis, Survival

## Abstract

**Background:**

Almost all of the extracellular matrix (ECM) components can be degraded by the endoproteinases matrix metalloproteinases (MMPs). Important regulators of MMPs, and thereby of the extracellular environment, are tissue inhibitors of metalloproteinases (TIMPs), and especially TIMP-1. Early tumor development, as well as distant metastasis, may be results of an MMP/TIMP ratio imbalance altering the ECM. MMPs are elevated in several inflammatory conditions. Our aim is to investigate the prognostic role of MMP-8, − 9, and TIMP-1 in colorectal cancer (CRC) and their relationship to inflammation.

**Methods:**

We included 337 colorectal cancer patients and 47 controls undergoing surgery at Helsinki University Hospital in Finland, 1998–2011. Serum levels of MMP-8 and plasma levels of C-reactive protein (CRP) were determined with a time-resolved immunofluorometric assay (IFMA), and MMP-9 and TIMP-1 with commercial enzyme-linked immunosorbent assay (ELISA) kits. Association and correlation analyses were performed with the Mann-Whitney U, Kruskal-Wallis, and Spearman rank correlation tests. Survival curves were constructed according to the Kaplan-Meier method and compared with the log-rank test.

**Results:**

Among patients with advanced disease, serum levels of MMP-8 and TIMP-1 were elevated. CRC patients with high MMP-8 (HR (hazard ratio) 1.72, 95% confidence interval (CI) 1.17–2.52, *P* = 0.005) and those with high TIMP-1 (HR 1.80, 95% CI 1.23–2.64, *P* = 0.002) had worse prognoses. MMP-9 level failed to serve as a prognostic factor. In multivariable survival analysis, Dukes stage, and low MMP-9/TIMP-1 molar ratio (HR 0.46, 95% CI 0.33–0.98, *P* = 0.042) were independently predicted prognosis. A weak correlation between CRP and MMP-8 (r_S_ = 0.229, *P* < 0.001), and TIMP-1 (r_S_ = 0.280, *P* < 0.001) was noted. Among patients showing no systemic inflammatory response, MMP-8 (HR 1.66, 95% CI 1.10–2.53, *P* = 0.017) and TIMP-1 (HR 1.59, 95% CI 1.05–2.42, *P* = 0.029) were prognostic factors.

**Conclusions:**

MMP-8 and TIMP-1 in serum, but not MMP-9, identified CRC patients with bad prognosis. Among patients showing no systemic inflammatory response, MMP-8 and TIMP-1 may associate with poor prognosis.

**Electronic supplementary material:**

The online version of this article (10.1186/s12885-018-4589-x) contains supplementary material, which is available to authorized users.

## Background

Colorectal cancer (CRC), one of the most common cancers, has a high morbidity level in developed countries [1, 2]. That survival has improved may be explained by increased screening or early awareness, as well as by improved treatment. Still, 17% of stage II and 36% of stage III patients develop a recurrence within 5 years [3]. What is of great importance is to recognize that these patients need adjuvant treatment and intensive follow-up, whereas others may be spared from laborious treatments.

Matrix metalloproteinases (MMPs) are a family of structurally related zinc-dependent endopeptidases capable of degrading almost all extraceullular matrix (ECM) components [4]. Increased MMP activity is a result of tumor cell-specific mechanisms such as angiogenesis and epithelial-mesenchymal transition (EMT). Additionally, MMPs can process distinct non-matrix bioactive substrates such as growth factors, complement components, pro- and anti-inflammatory cytokines, chemokines, serum proteins, and receptors. MMPs can thereby regulate immuneresponses [5, 6].

Of the 26 different MMPs recognized, several have been studied extensively in inflammatory diseases and cancer. MMP-8 and -9 belong to the collagenase subgroup of MMPs being expressed – apart from tumor cells – also by fibroblasts and infiltrating inflammatory cells [7]. In benign tissues, the ECM environment is strictly under the control, among others, of endogenous proteins called tissue inhibitors of metalloproteinases (TIMPs) [8]. TIMP-1 in particular serves by binding to MMPs as an important regulator and may inhibit the proteolytic activity of MMPs. Any imbalances in this strictly controlled process may result in altered ECM and early tumor development, and a disruption of the MMP/TIMP ratio within the microenvironment may facilitate distant metastasis [9]. TIMP-1 also exerts MMP-inhibition-independent characteristics such as growth factor-like and proinflammatory properties [10].

Elevated levels of serum MMP-8, MMP-9, and TIMP-1 have appeared in several cancers: lung, gastric, hepatocellular, and colorectal, but also in melanoma and head and neck cancer [11–17]. MMP-9 in particular has been extensively studied for its effects on tumor cell invasion and angiogenesis. The influence of elevated MMP-9 serum level on prognosis and on its ability to serve as a diagnostic maker have, however, varied [7]. MMP-8, on the other hand, has not raised as much interest, although it regulates many different proteins of the ECM [18]. High serum MMP-8 level correlates with stage, but its effect on survival was not reported there [14]. Some have shown that in colorectal cancer, preoperative plasma TIMP-1 serves as an independent prognostic marker [19], whereas others found only a limited value for TIMP-1 as a prognostic indicator [20]. According to a meta-analysis based on five different studies, colorectal cancer patients with elevated plasma or serum TIMP-1 had poorer overall survival [21]. As the balance between MMPs and TIMP-1 is tightly regulated in healthy tissues, their molar ratio may more specifically reflect the ECM environment in malignant lesions.

Colorectal cancer patients showing systemic inflammatory response have a worse prognosis [22]. In multiple other conditions linked to an activated inflammatory response, such as acute coronary syndrome, chronic urticaria, or pancreatitis, correlations exist between high MMP-9 levels and C-reactive protein (CRP) [23–25]. In colorectal cancer, high serum MMP-8 levels and high blood neutrophil and leukocyte count correlated positively [14], but correlations between TIMP-1 and white blood cell count were less clear.

The aim of our study was to investigate the prognostic roles of MMP-8, MMP-9, and TIMP-1 in colorectal cancer. Furthermore, we studied any possible relationship between elevated MMP-levels and systemic inflammatory response.

## Methods

### Patients

Of 384 patients undergoing surgery at Helsinki University Hospital, Finland, 1998–2011, 335 underwent a primary elective operation for colorectal cancer, and 47 with surgery for other reasons served as benign controls. Colorectal cancer (CRC) patients had surgery in 1998–2003 with a median follow-up time of 6.4 years (range, 1 day to 16.3 years). At the end of follow-up, 200 (59.3%) had died. The 5-year disease-specific survival for colorectal cancer patients was 69.9% (95% confidence interval (CI) 64.6–75.2), for colon cancer patients, it was 72.1% (95% CI 64.5–79.5), and for rectal cancer patients, 67.4% (95% CI 60.3–75.1). Of the CRC patients, 173 (51.3%) were men, and 257 (76.3%) had surgery with curative intent. In 156 (46.6%) patients and the tumor was situated in the colon and in 179 (53.4%) in the rectum; it was more frequently located in the left side of the colorectum (242; 72.2%) (Table 1).

**Table 1:** Characteristics of 335 colorectal cancer patients

Patient characteristics	n (%)
Age	
Median (IQR), years	67.2 (57.5–75.9)
Gender	
Men	174 (51.9)
Women	161 (48.1)
Dukes classification	
A	59 (17.6)
B	101 (30.1)
C	114 (34.0)
D	61 (18.2)
Tumor classification (pT)	
pT1	13 (3.9)
pT2	74 (22.1)
pT3	212 (63.3)
pT4	31 (9.3)
Lymph node metastasis (pN)	
pN0	176 (52.5)
pN1	87 (26.0)
pN2	68 (20.3)
Distant metastasis (pM)	
pM0	273 (81.5)
pM1	58 (17.3)
Grade (WHO)	
1	22 (6.6)
2	31 (69.0)
3	24 (7.2)
4	22 (6.6)
Location	
Colon	156 (46.6)
Rectum	179 (53.4)
Side	
Right	93 (27.8)
Left	242 (72.2)
Histologic type	
Adeno	309 (92.2)
Mucinous	26 (7.8)
Systemic inflammatory response	
CRP < =30	278 (83.0)
CRP > 30	51 (15.2)

*Abbreviation*: *IQR* interquartile range

Median age was for the 47 controls 54.0 (interquartile range (IQR) 38.5–70.9), and 30 (64.8%) were women. They underwent surgery for benign colorectal neoplasia (18; 38.3%), inflammatory bowel disease (13; 27.7%), or benign thyroid disease (11; 23.4%), and the other 5 (10.6%) for other reasons. Their 5-year overall survival was 90.3% (95% CI 81.2–99.3).

### Serum and plasma samples

Blood samples were obtained within 30 days prior to surgery (range 0–30 days). The majority of the samples (92.4%) were taken within 3 days preoperatively. The samples were centrifuged, and serum and plasma components stored as aliquots at − 80 **°**C until analysis. The commercial MMP-9 and TIMP-1 enzyme-linked immunosorbent assay (ELISA) kits served for determination of serum levels in accordance with the manufacturer’s instructions (Biotrak ELISA System; Amersham Biosciences, Buckinghamshire, UK). For MMP-9, the detection limit was 0.6 ng/ml and for TIMP-1 1.25 ng/ml [6]. For MMP-8, we used the time-resolved immunofluorometric assay (IFMA) (Medix Biochemica, Espoo, Finland) in accordance with the manufacturer’s instructions with a detection limit of 0.08 ng/ml [26].

We determined plasma CRP by a high-sensitivity method; time-resolved IFMA, with a monoclonal CRP antibody (anti-hCRP, code 6405, Medix Biochemica) as previously described [27].

### Statistical analysis

To determine the significance of the difference in biomarker concentrations, the Mann-Whitney U-test and Kruskal-Wallis test were applied. Correlations between the biomarkers and CRP were explored by the Spearman rank correlation test. We counted disease-specific survival from date of surgery to date of death from colorectal cancer or until end of follow-up. We used the Kaplan-Meier method to construct survival curves and compared them with the log-rank test. For biomarkers MMP-8, MMP-9, TIMP-1, MMP-8/TIMP-1, their molar ratios, and the MMP-9/TIMP-1 molar ratio were grouped as low or high according to their median values for survival analyses. For CRP, a concentration of ≤30 mg/l served as the cut-off for dichotomization. The Cox proportional hazard model served for multivariable survival analysis and we entered the following covariates: gender, age, Dukes stage, grade, histologic type, tumor location (colon vs. rectum), side (right vs. left), MMP-8, − 9, TIMP-1, and CRP serum concentration, as well as MMP/TIMP-1 molar ratios. Dukes’ classification and grade, were entered as categorical covariates. Multivariable Cox regression analysis was performed according to the backward stepwise method with removal of the term at *P* < 0.1. Interaction terms were considered in the final model, with no significant interactions found. The Cox proportional hazard model assumption of constant hazard ratios over time was tested by including a time-dependent variable for each testable variable separately. All variables fulfilled the assumption. We considered *P*-values of < 0.05 statistically significant. We used the IBM SPSS Statistics version 23.0 for Mac (IBM Corporation, Armonk, NY, USA) for the statistical analyses.

## Results

Of median MMP-8, MMP-9, and TIMP-1 serum levels prior to surgery for colorectal cancer and for controls with benign disease, only TIMP-1 levels were higher in patients with CRC than in controls (*P* = 0.037, Mann-Whitney U-test, Table 2). No differences in molar ratios of MMP/TIMP-1 were noted between cancer patients and controls. Colorectal cancer patients had higher CRP levels than did controls (*P* < 0.001).

**Table 2:** Median serum concentrations of MMP-8, MMP-9, TIMP-1, and molar ratios of MMPs and TIMP-1 in 335 colorectal cancer and 47 control patients

	Colorectal cancer		Controls		
	Median	IQR	Median	IQR	P-value^a^
MMP-8 (ng/ml)	60	33–118	68	39–107	0.358
MMP-9 (ng/ml)	192	123–273	159	60–252	0.216
TIMP-1 (ng/ml)	151	131–180	139	125–161	0.037
MMP-8/TIMP-1 (molar ratio)	0.158	0.094–0.314	0.208	0.130–0.345	0.109
MMP-9/TIMP-1 (molar ratio)	0.368	0.216–0.539	0.343	0.139–0.610	0.755
CRP (mg/l)	4.85	1.92–15.6	1.21	0.404–4.14	< 0.001

*Abbreviations*: *MMP* matrix metalloproteinase, *TIMP-1* tissue inhibitor of metalloproteinases-1, *CRP* C-reactive protein, *IQR* interquartile range

^a^Mann-Whitney U-test

### Association of MMP-8, MMP-9, and TIMP-1 with clinicopathologic parameters

Serum levels of MMP-8 were higher among patients with advanced disease, both in regard to locally advanced (pT4 tumors; *P* = 0.004) and distantly metastasized disease (*P* < 0.001, Table 3). Serum MMP-8 was also higher among those with the tumor located in the right side of the colon (*P* = 0.038). Serum MMP-9 levels were slightly higher in men (*P* = 0.015) and in those with metastasized disease *(P* = 0.028). TIMP-1 serum levels were likewise higher among patients with locally advanced disease (pT4 tumors; *P* = 0.028), as well as higher among patients with a right-sided tumor (*P* = 0.016). In addition, serum TIMP-1 was higher among patients over 65 (*P* < 0.001).

**Table 3:** Significance of the difference in MMP-8, −9, and TIMP-1 serum concentrations in 330 colorectal cancer patients

Clinicopathological variable	MMP-8		MMP-9		TIMP-1	
	Median (IQR)	*P*-value	Median (IQR)	*P*-value	Median (IQR)	*P*-value
Age^a^						
< =65	56 (36–97)	0.773	205 (136–287)	0.159	138 (123–162)	< 0.001
> 65	63 (32–125)		185 (123–265)		164 (141–196)	
Gender^a^						
Male	60 (32–122)	0.861	209 (141–299)	0.015	154 (135–185)	0.020
Female	57 (35–112)		183 (105–254)		147 (123–177)	
Dukes classification^b^						
A	50 (31–76)	< 0.001	171 (95–269)	0.040	142 (122–163)	0.024
B	55 (33–90)		194 (131–318)		153 (131–183)	
C	51 (30–101)		183 (123–246)		152 (134–178)	
D	137 (56–328)		223 (161–310)		166 (132–257)	
pT^b^						
pT1	50 (22–64)	0.004	237 (83–297)	0.113	144 (117–190)	0.028
pT2	50 (27–90)		164 (101–244)		144 (126–166)	
pT3	58 (34–129)		195 (128–287)		152 (131–179)	
pT4	93 (58–265)		211 (165–284)		180 (148–244)	
pN^b^						
pN0	56 (33–93)	0.238	148 (127–175)	0.183	149 (127)	0.372
pN1	64 (33–93)		154 (135–181)		154 (135–181)	
pN2	63 (35–140)		160 (130–188)		160 (130–188)	
pM^a^						
pM0	54 (32–94)	< 0.001	189 (121–265)	0.028	150 (131–175)	0.054
pM1	118 (56–332)		221 (159–304)		165 (131–259)	
Grade (WHO)^b^						
1	66 (34–173)	0.227	204 (137–293)	0.717	166 (110–197)	0.727
2	60 (33–113)		193 (116–273)		150 (131–179)	
3	41 (27–78)		159 (136–299)		163 (137–182)	
4	102 (36–185)		215 (183–273)		157 (126–189)	
Location^a^						
Colon	65 (35–134)	0.104	204 (143–284)	0.116	157 (131–187)	0.053
Rectum	54 (32–103)		185 (109–273)		148 (131–174)	
Side^a^						
Right	70 (39–134)	0.038	208 (152–290)	0.266	161 (135 (191)	0.016
Left	54 (32–104)		189 (120–273)		149 (130–175)	
Histologic type^a^						
Adeno	58 (33–120)	0.726	192 (125–274)	0.374	152 (131–182)	0.558
Mucinous	63 (40–96)		221 (157–320)		156 (126–198)	

*Abbreviations*: *MMP* matrix metalloproteinase, *TIMP-1* tissue inhibitor of matrix metalloproteinase-1, *IQR* interquartile range

^a^Mann-Whitney U-test, ^b^Kruskal-Wallis test

The MMP-8/TIMP-1 molar ratio was as well higher among patients with metastasized disease (*P* < 0.001, Additional file 1). The MMP-9/TIMP-1 molar ratio was higher among patients under 65 (*P* = 0.002).

We found weak positive correlations between MMP-8 and CRP (r_S_ = 0.229, *p* < 0.001, Spearman rank correlation test), between TIMP-1 and CRP (r_S_ = 0.280, *P* < 0.001), and between MMP-8/TIMP-1 molar ratio and CRP (r_S_ = 0.151, *P* = 0.007). No significant correlation was noted between MMP-9 and CRP (r_S_ = 0.110, *P* = 0.050) or MMP-9/TIMP-1 molar ratio and CRP (r_S_ = − 0.023, *P* = 0.678).

### Univariable survival analyses

**Fig. 1:**
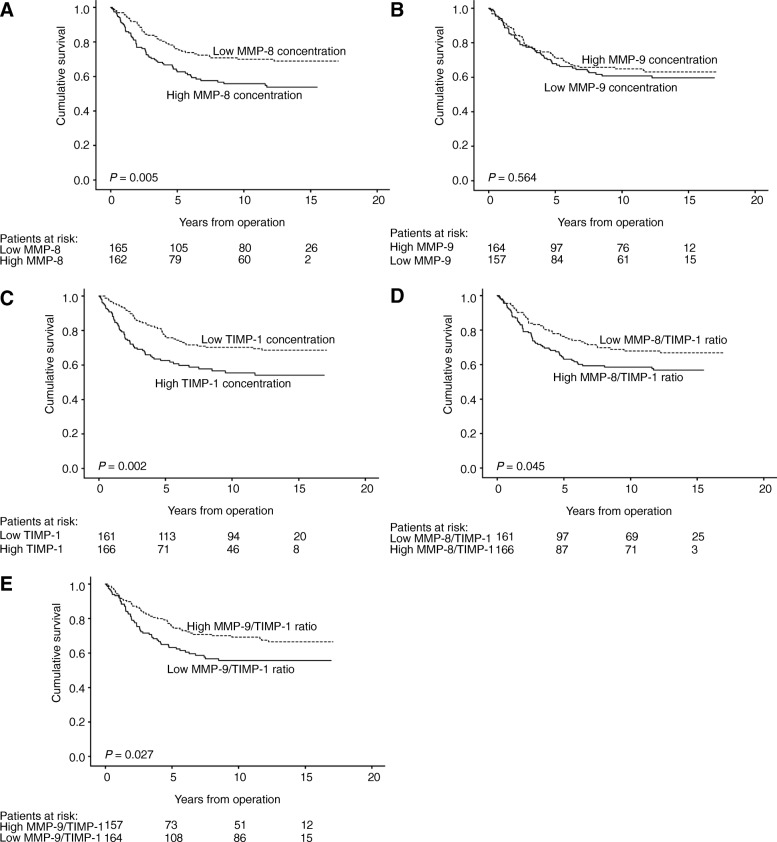
Disease-specific survival in colorectal cancer patients according to the Kaplan-Meier method. Low vs. high expression in serum of (**a**) MMP-8, (**b**) MMP-9, (**c**) TIMP-1, (**d**) MMP-8/TIMP-1 molar ratio, and (**e**) MMP-9/TIMP-1 molar ratio. *P*-value for log-rank test

Five-year disease-specific survival according to dichotomized MMP-8, − 9, and TIMP-1 concentrations and MMP/TIMP-1 molar ratios are in Additional file 2 and univariable hazard ratios in Table 4. Colorectal cancer patients with low MMP-8 levels had a 5-year survival of 76.0% (95% CI 69.1–82.9) and those with high MMP-8 levels 62.7% (95% CI 54.7–70.7; HR (hazard ratio) 1.72, 95% CI 1.17–2.52, *P* = 0.005; Fig. 1). Patients with low TIMP-1 levels had a 5-year survival of 76.4% (95% CI 69.7–83.1) and those with high TIMP-1 levels 62.6% (95% CI 54.6–70.6; HR 1.80, 95% CI 1.23–2.64, *P* = 0.002). Patients with high MMP-8/TIMP-1 molar ratio had better survival (HR 1.48, 95% CI 1.0–2.16, *P* = 0.045), whereas patients with a low MMP-9/TIMP-1 molar ratio survived longer (HR 0.65, 95% CI 0.45–0.96, *P* = 0.027; Additional file 2 and Table 4). MMP-9 level did not serve as a prognostic factor.

**Table 4:** Univariable Cox regression analysis of disease-specific survival for colorectal cancer patients

	Hazard ratio	95% CI	*P*-value
Age, years			
< =65	1.00		
> 65	1.44	0.98–2.10	0.062
Gender			
Male	1.00		
Female	1.06	0.73–1.54	0.772
Dukes classification			
A	1.00		
B	3.33	0.96–11.5	0.057
C	11.0	3.45–35.3	< 0.001
D	29.3	9.05–95.1	< 0.001
pT stage			
pT1	1.00		
pT2	1.62	0.21–12.8	0.648
pT3	6.33	0.88–45.5	0.067
pT4	9.89	1.31–75.0	0.067
pN stage			
pN0	1.00		
pN1	3.77	2.33–6.09	< 0.001
pN2	5.16	3.15–8.50	< 0.001
pM stage			
pM0	1.00		
pM1	5.31	3.55–7.93	< 0.001
Grade			
I	1.00		
II	1.38	0.60–3.17	0.445
III	1.07	0.36–3.18	0.906
IV	2.16	0.78–5.93	0.137
Side			
Right	1.00		
Left	1.45	0.92–2.28	0.107
Location			
Colon	1.00		
Rectum	1.16	0.79–1.69	0.453
Histologic type			
Adeno	1.00		
Mucinous	0.95	0.46–1.95	0.888
MMP-8 concentration			
Low	1.00		
High	1.72	1.17–2.52	0.005
MMP-9 concentration			
Low	1.00		
High	0.89	0.61–1.31	0.564
TIMP-1 concentration			
Low	1.00		
High	1.80	1.23–2.64	0.002
MMP-8/TIMP-1 ratio			
Low	1.00		
High	1.48	1.01–2.16	0.045
MMP-9/TIMP-1 ratio			
Low	1.00		
High	0.65	0.45–0.96	0.027
CRP (mg/l)			
< =30	1.00		
> 30	1.75	1.09–2.82	0.021

*Abbreviations*: *MMP* matrix metalloproteinase, *TIMP-1* tissue inhibitor of metalloproteinases-1, *CI* confidence interval

**Fig. 2:**
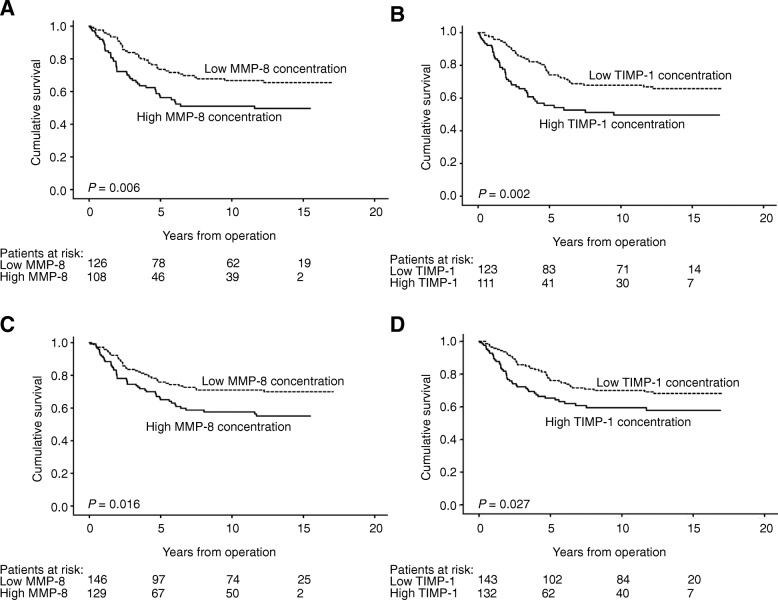
Disease-specific survival of subgroups of colorectal cancer patients according to the Kaplan-Meier method. In patients with left-sided disease, low vs. high serum levels of (**a**) MMP-8 and (**b**), TIMP-1. In patients showing no systemic inflammatory response (CRP < 30 mg/l), low vs. high serum levels of (**c**) MMP-8 and (**d**), TIMP-1. *P*-value for log-rank test

In subgroup analyses, survival was poor for patients with high MMP-8 and colon cancer (HR 2.00, 95% CI 1.10–3.64, *P* = 0.023, Additional file 3), with left-sided tumor (HR 1.80, 95% CI 1.17–2.77, *P* = 0.007), and with no systemic inflammatory response (HR 1.66, 95% CI 1.10–2.53, *P* = 0.017, Fig. 2a-b). Low levels of MMP-9 indicated poor prognosis among rectal cancer patients (HR 0.49, 95% CI 0.28–0.85, *P* = 0.011; Additional file 3). High TIMP-1 levels indicated poor survival among patients with rectal cancer (HR 1.95, 95% CI 1.17–3.26, *P* = 0.011), with left-sided tumor (HR 1.95, 95% CI 1.27–3.00, *P* = 0.002), and with low CRP (HR 1.59, 95% CI 1.05–2.42, *P* = 0.029, Fig. 2c-d).

### Multivariable survival analysis

We found that age, Dukes stage, and low MMP-9/TIMP-1 molar ratio (HR 0.46, 95% CI 0.33–0.98, *P* = 0.042) served as independent prognostic factors (Table 5).

**Table 5:** Multivariable Cox regression analysis of disease-specific survival for colorectal cancer patients

	Hazard ratio	95% CI	*P*-value
Age, years			
< =65	1.00		
> 65	1.86	1.14–3.03	0.013
Dukes classification			
A	1.00		
B	1.87	0.504–6.93	0.350
C	8.63	2.64–28.2	< 0.001
D	15.1	4.43–51.6	< 0.001
MMP-8/TIMP-1 ratio			
Low	1.00		
High	1.72	0.979–3.02	0.059
MMP-9/TIMP-1 ratio			
Low	1.00		
High	0.573	0.335–0.980	0.042

*Abbreviations*: *MMP* matrix metalloproteinase, *TIMP-1* tissue inhibitor of metalloproteinases-1, *CI* confidence interval

## Discussion

In colorectal cancer, we found that high levels of serum MMP-8 and TIMP-1 serve as prognostic factors. Interestingly, serum MMP-9 did not influence prognosis, but low MMP-9/TIMP-1 molar ratio, together with high age and advanced Dukes stage, were each independent prognostic factors for poor prognosis. We noted that high MMP-8 and TIMP-1 associated with advanced stage and right-sided location. Among patients with normal CRP, indicating lack of systemic inflammatory response, high MMP-8 and TIMP-1 selected patients with poor prognosis.

Few studies concern the prognostic value of MMP-8, MMP-9, or TIMP-1 in colorectal cancer. In a study with 148 colorectal cancer patients, high serum MMP-8 and TIMP-1 associated with advanced stage [14]. In another, with 97 colon cancer patients, high TIMP-1 associated with shorter overall survival and emerged as an independent prognostic factor [28]. We also found an association between advanced stage (Dukes D) and high serum MMP-8 and TIMP-1. Among patients with metastasized disease, MMP-8 and TIMP-1 were more commonly elevated; their levels were higher among those with right-sided disease, which has not been previously reported. We measured MMP-9 and TIMP-1 serum levels by ELISA and MMP-8 with an IFMA method, in contrast to that of with another group, which utilized gelatin-zymography (SDS-PAGE) to detect MMP-9 levels [15]. In their 32 colorectal cancer patients, MMP-2 and MMP-9 seemed to correlate with more advanced stage; however, by this method, their results may be, at least in part, uncertain [15]. Gelatin-zymography assaying semiquantitatively SDS-treated catalytic activities of MMP-2 and -9 does not analyze the concentrations of MMP-2 and -9 as precisely as do IFMA and ELISA utilizing highly specific antibodies [29]. Their conclusion that MMP-9 would serve as an independent prognostic marker cannot be drawn based on their results. All in all, MMP-8 and TIMP-1 seem to influence the prognosis of colorectal cancer patients to a greater extent than MMP-9 seems to do.

In hepatocellular carcinoma, high levels of MMP-8 and TIMP-1 have indicated poor survival, as did our levels in CRC [13]. We found in CRC that although MMP-9 levels had no influence on survival, patients with a low MMP-9/TIMP-1 ratio had impaired survival, in line with their results in hepatocellular carcinoma. Similar findings have appeared also in patients with malignant melanoma, for whom high TIMP-1 indicated impaired disease-free survival [16], and in head and neck squamous cell carcinoma patients who had shorter disease-free survival [17].

Prognosis is worse for patients with right-sided colorectal (RCC) than with left-sided colorectal cancer (LCC) [30]. Right-sided tumors more frequently are microsatellite instable and, express *KRAS* and *BRAF* mutations, whereas LCC patients more frequently have mutations in p53, *NRAS*, and show chromosomal instability (CIN) which may imply a different genetic background [31, 32]. Moreover, patients with RCC microsatellite stable tumors have a significantly worse prognosis than those that have microsatellite instable tumors [31]. Although MMP-8 and TIMP-1 expression more frequently was higher in patients with right-sided tumors, MMP-8 and TIMP-1 did not serve as prognostic factors among these subgroups. On the contrary, we found that high MMP-8 served as a prognostic factor in the subgroup of colon cancer and patients with tumors located on the left side of the colorectum.

Among colon cancer patients, especially within those with left-sided disease, MMP-8 served as a prognostic factor. In rectal cancer, high MMP-9 and high TIMP-1 served as prognostic factors. In addition, high TIMP-1 was an indicator of poor prognosis among patients with left-sided disease. These acknowledged differences in genetic background explain why MMP expression influences prognosis in different ways. Instead of the two-sided colon model – where right and left are divided at the splenic flexure – a shift towards a multisegmental model displaying a continual shift in protein expression may eventually better serve as a model [32].

CRP is a marker of systemic inflammation, with a convincing prognostic influence in colorectal cancer [22, 33]. As on one hand, TIMP-1 has a growth factor-like role directly affecting cancer cell growth, invasion, and migration independent of TIMP:s inhibition of MMPs [9, 10], and on the other hand, both MMPs and TIMP-1 play an important role in inflammatory processes, we explored whether MMPs and CRP correlate. We found a weak positive correlation between MMP-8 and CRP levels, as well as between TIMP-1 and CRP. In patients with low CRP, high MMP-8 and TIMP-1 were prognostic factors independent of CRP, whereas among patients with high CRP reflecting a systemic inflammation response, MMPs or TIMP-1 failed to select those patients with a worse prognosis. In acute coronary syndrome, MMP and CRP are correlated, and CRP seems to induce local MMP-9 secretion [25]. In chronic urticaria, on the other hand, high levels of MMP-9 and CRP are related to disease severity [23]. Oral cancer patients with high MMP-9 and CRP levels have had worse prognosis [34].

Few studies have reported on the relation between MMP and CRP in CRC. During adenoma-carcinoma development, a progressive increase in interleukin-8 (IL-8), CRP, and MMP-9 occurs. Among 26 stage III colorectal cancer patients, levels of MMP-9 and IL-8 were significantly elevated and correlated with each other [35]. Likewise, MMP-8 correlates with high leukocyte and neutrophil count, whereas TIMP-1 correlates only weakly with neutrophil count [14]. Among 525 colon cancer cases, patients with high CRP had poor prognosis, a result that was sustained within all disease stages when analyzed separately [22]. Kostner et al. [33] demonstrated that even among CRC patients with metastatic disease, high CRP serves as a prognostic marker. MMP-8 is produced mainly by neutrophils and reflects a response in the acute phase of inflammation [5, 36]. Conversely, MMP-9 is produced by several different cell types, so its serum levels are more easily affected and any correlation with stage or prognosis may thus be diluted [4].

We found that, compared with levels in healthy controls, only TIMP-1 levels were marginally elevated among the cancer patients. In contrast, in a study on 180 patients, of whom 75 had colorectal cancer, MMP-9 and TIMP-1 levels were higher in colorectal cancer patients than in healthy controls or in colorectal adenoma patients [37]. In that study, MMP-9 and TIMP-1 also correlated with tumor stage, but no data were presented on survival nor, in contrast with our results, did patients with colon cancer have higher levels of MMP-9 than did rectal cancer patients. Our primary aim was to study the prognostic role of MMP-8, − 9, and TIMP-1, and thus, benign control patients could be few.

We investigated optimal cut-offs by the aid of receiver-operating curves (ROC) and found them to be close to median values. Therefore, we chose to dichotomize our variables according to median values. Unfortunately, it was impossible to study the correlation between serum and tissue expression of MMP-8 and -9, because the previous tissue results were from an earlier series studied at our institution [38]. In that series, we found that MMP-9 served as a prognostic marker among Dukes B patients. However, protein serum and tissue expression does not necessarily correlate; local expression in tissue may reflect intact cells and glands, and hence only small amounts of the protein are released into the circulation.

## Conclusions

Serum MMP-8 and TIMP-1 may serve as prognostic factors in colorectal cancer. Among patients showing no systemic inflammatory response, high MMP-8 and TIMP-1 may associate with poor prognosis.

## Additional files

Additional file 1: Significance of the difference in MMP-8/TIMP-1 and MMP-9/TIMP-1 molar ratios in 330 colorectal cancer patients. (PDF 27 kb)

Additional file 2: 5-year disease-specific survival with 95% confidence intervals for MMP-8, − 9, and TIMP-1 in colorectal cancer patients. (PDF 20 kb)

Additional file 3: Univariable Cox regression analyses for subgroups for disease-specific survival in colorectal cancer patients. (PDF 19 kb)

## Abbreviations

CI: Confidence interval
CIN: Chromosomal instability
CRC: Colorectal cancer
CRP: C-reactive protein
ELISA: Enzyme-linked immunosorbent assay
HR: Hazard ratio
IFMA: Immunofluorometric assay
IL-8: Interleukin-8
IQR: Interquartile range
LCC: Left-sided colorectal cancer
MMP: Matrix metalloproteinase
RCC: Right-sided colorectal cancer
SDS-PAGE: Sodium dodecyl sulfate-polyacrymid gel
TIMP-1: Tissue inhibitor of metalloproteinases-1
