# Supplementary material for: Serum MMP-8 and TIMP-1 predict prognosis in colorectal cancer
Source: BMC Cancer. 2018 Jun 22;18:679. doi: 10.1186/s12885-018-4589-x (PMC6013876; doi:10.1186/s12885-018-4589-x)
Supplement: Supplementary file 1 — Significance of the difference in MMP-8/TIMP-1 and MMP-9/TIMP-1 molar ratios in 330 colorectal cancer patients. (PDF 27 kb) [file 12885_2018_4589_MOESM1_ESM.pdf]

Additional file 1. Significance of the difference in MMP-8/TIMP-1 and MMP-9/TIMP-1 molar ratios in 330 colorectal cancer patients.

**Clinicopathological variable**	**MMP-8/TIMP-1**		**MMP-9/TIMP-1**	
	Median (IQR)	*P*-value	Median (IQR)	*P*-value
Age[1]				
<=65	0.189 (0.099-0.320)	0.179	0.429 (0.260-0.625)	0.002
>65	0.153 (0.089-0.315)		0.332 (0.210-0.475)	
Gender[1]				
Male	0.157 (0.092-0.306)	0.530	0.389 (0.240-0.573)	0.170
Female	0.172 (0.097-0.321)		0.356 (0.193-0.517)	
Dukes classification[2]				
A	0.142 (0.093-0.261)	0.001	0.349 (0.229-0.609)	0.675
B	0.154 (0.085-0.255)		0.408 (0.251-0.550)	
C	0.151 (0.093 (0.276)		0.357 (0.216-0.498)	
D	0.305 (0.142-0.659)		0.366 (0.188-0.597)	
pT[2]				
pT1	0.145 (0.067-0.167)	0.169	0.349 (0.165-0.815	0.612
pT2	0.136 (0.091-0.281)		0.334 (0.223-0.533)	
pT3	0.173 (0.097-0.330)		0.383 (0.228-0.552)	
pT4	0.199 (0.133-0.366)		0.396 (0.261-0.500)	
pN[2]				
pN0	0.156 (0.093-0.273)	0.379	0.380 (0.232-0.563)	0.583
pN1	0.180 (0.096-0.384)		0.349 (0.219-0.478)	
pN2	0.179 (0.101-0.352)			
pM[1]				
pM0	0.152 (0.093-0.272)	<0.001	0.370 (0.230-0.527)	0.821
pM1	0.300 (0.143-0.646)		0.379 (0.184-0.617)	
Grade (WHO)[2]				
1	0.164 (0.082-0.442)	0.174	0.356 (0.256-0.503)	0.604
2	0.159 (0.093-0.313)		0.368 (0.212-0.569)	
3	0.101 (0.069-0.200)		0.294 (0.231-0.481)	
4	0.201 (0.117-0.411)		0.449 (0.315-0.603)	
Location[1]				
Colon	0.176 (0.101-0.322)	0.127	0.389 (0.253-0.539)	0.313
Rectum	0.155 (0.092-0.300)		0.344 (0.192-0.567)	
Side[1]				
Right	0.189 (0.104-0.330)	0.109	0.370 (0.252-0.524)	0.882
Left	0.154 (0.092-0.302)		0.379 (0.210-0.563)	
Histologic type[1]				
Adeno	0.162 (0.093-0.317)	0.748	0.369 (0.217-0.555)	0.427
Mucinous	0.158 (0.121-0.352)		0.435 (0.298-0.508)	

Additional file 1. Significance of the difference in MMP-8/TIMP-1 and MMP-9/TIMP-1 molar ratios in 330 colorectal cancer patients.

Abbreviations: MMP = matrix metalloproteinase, TIMP-1 = tissue inhibitor of matrix metalloproteinase-1, IQR = interquartile range
[1]Mann-Whitney U test, [2]Kruskal-Wallis test
