# Supplementary material for: Serum MMP-8 and TIMP-1 predict prognosis in colorectal cancer
Source: BMC Cancer. 2018 Jun 22;18:679. doi: 10.1186/s12885-018-4589-x (PMC6013876; doi:10.1186/s12885-018-4589-x)
Supplement: Supplementary file 2 — 5-year disease-specific survival with 95% confidence intervals for MMP-8, − 9, and TIMP-1 in colorectal cancer patients. (PDF 20 kb) [file 12885_2018_4589_MOESM2_ESM.pdf]

Additional file 2. 5-year disease-specific survival with 95% confidence intervals for MMP-8, -9, and TIMP-1 in colorectal cancer patients

Biomarker concentration	Low	High
MMP-8	76.0 (69.1-82.9)	62.7 (54.7-70.7)
MMP-9	67.8 (60.0-75.6)	71.0 (63.7-78.3)
TIMP-1	76.4 (69.7-83.1)	62.6 (54.6-70.6)
MMP-8/TIMP-1 ratio	76.4 (69.3-83.5)	63.2 (55.4-71.0)
MMP-9/TIMP-1 ratio	63.2 (55.0-71.4)	75.1 (68.2-82.0)

Abbreviations: MMP = matrix metalloproteinase, TIMP-1 = tissue inhibitor of metalloproteinases-1, CI = confidence interval

[1]Log-rank test
