# Supplementary material for: Serum MMP-8 and TIMP-1 predict prognosis in colorectal cancer
Source: BMC Cancer. 2018 Jun 22;18:679. doi: 10.1186/s12885-018-4589-x (PMC6013876; doi:10.1186/s12885-018-4589-x)
Supplement: Supplementary file 3 — Univariable Cox regression analyses for subgroups for disease-specific survival in colorectal cancer patients. (PDF 19 kb) [file 12885_2018_4589_MOESM3_ESM.pdf]

**Additional file 3.** Univariable Cox regression analyses for subgroups for disease-specific survival in colorectal cancer patients.

| | High MMP-8 | | | High MMP-9 | | | High TIMP-1 | | |
|---|---|---|---|---|---|---|---|---|---|
| | HR | 95% CI | P-value | HR | 95% CI | P-value | HR | 95% CI | P-value |
| Location | | | | | | | | | |
| Colon | 2.00 | 1.10-3.64 | 0.023 | 1.84 | 1.00-3.38 | 0.050 | 1.78 | 0.99-3.19 | 0.052 |
| Rectum | 1.62 | 0.97-2.70 | 0.065 | 0.49 | 0.28-0.85 | 0.011 | 1.95 | 1.17-3.26 | 0.011 |
| Side | | | | | | | | | |
| Right | 1.92 | 0.79-4.62 | 0.148 | 1.67 | 0.72-3.91 | 0.235 | 1.83 | 0.78-4.28 | 0.166 |
| Left | 1.80 | 1.17-2.77 | 0.007 | 0.76 | 0.49-1.16 | 0.203 | 1.95 | 1.27-3.00 | 0.002 |
| CRP level | | | | | | | | | |
| <30 | 1.66 | 1.10-2.53 | 0.017 | 0.79 | 0.52-1.20 | 0.267 | 1.59 | 1.05-2.42 | 0.029 |
| >=30 | 1.60 | 0.58-4.44 | 0.369 | 1.77 | 0.72-4.37 | 0.216 | 2.92 | 0.96-8.82 | 0.058 |

Abbreviations: MMP = matrix metalloproteinase, TIMP-1 = tissue inhibitor of matrix metallo-proteinases, CRP = C-reactive protein, HR = hazard ratio, CI = confidence interval
